# Altered functional connectivity density in the brains of hemodialysis end-stage renal disease patients: An *in vivo* resting-state functional MRI study

**DOI:** 10.1371/journal.pone.0227123

**Published:** 2019-12-31

**Authors:** Yan Shi, Chaoyang Tong, Minghao Zhang, Xiaoling Gao

**Affiliations:** 1 Department of Nephrology, The Ninth People’s Hospital of Chongqing, Chongqing, China; 2 Department of Medical Imaging, The Ninth People’s Hospital of Chongqing, Chongqing, China; 3 Center for Lab Teaching and Management, Chongqing Medical University, Chongqing, China; University of Texas at Austin, UNITED STATES

## Abstract

**Background:**

End-stage renal disease (ESRD) patients usually suffer from a high prevalence of central nervous system abnormalities, including cognitive impairment and emotional disorders, which severely influence their quality of life. There have been many neuroimaging research developments in ESRD patients with brain function abnormalities; however, the dysfunction of the salience network (SN) of them has received little attention. The purpose of this study was to investigate the changes of global functional connectivity density (gFCD) in brains of ESRD patients undergoing hemodialysis using resting-state functional magnetic resonance imaging (re-fMRI).

**Methods:**

re-fMRI data were collected from 30 ESRD patients undergoing hemodialysis (14 men, 38.33±7.44 years old) and 30 matched healthy controls (13 men, 39.17±5.7 years old). Neuropsychological tests including the Montreal Cognitive Assessment (MoCA) and Beck Depression Inventory (BDI) were used to evaluate the neurocognitive and psychiatric conditions of the subjects. Blood biochemistry tests, including hemoglobin level, serum albumin level, blood urea level, serum phosphate, serum calcium, and parathyroid hormone level, and dialysis-related indicators, including blood pressure fluctuations in dialysis, single-pool Kt/V(spKt/V), and ultrafiltration volume of dialysis were obtained from the ESRD patients. A two-sample t-test was used to examine the group differences in gFCD between ESRD patients and healthy controls after controlling for age, gender and education.

**Results:**

Compared with healthy controls, ESRD patients exhibited a significantly increased gFCD in the salience network, including the bilateral insula, and dorsal anterior cingulated cortex (dACC), and there was no significant correlation between gFCD and the structural mean grey matter volume in patients for every cluster in the brain regions showing significant different gFCD between the two groups. Furthermore, there were significant negative correlations between the degree of connectivity in the right insula and spKt/V.

**Conclusion:**

Our findings revealed abnormal intrinsic dysconnectivity pattern of salience network-related regions in ESRD patients from the whole brain network perspective. The negative correlation between the right insula and spKt/V suggested that increased fractional removal of urea may reduce the pathological activity in the insula.

## Introduction

End-stage renal disease (ESRD) is the last stage of chronic kidney disease, and it has shown a remarkable increase with the aging of the population and the rise of chronic disease in recent years. Along with a high prevalence of cerebral lesions [1–4], patients with ESRD also suffer from an increased risk of neurological dysfunction, such as cognitive impairment, and psychological disorders such as depression and anxiety [5,6], which contribute to non-adherence to dialysis and medication compliance and lower quality of life, and lead to more frequent hospitalization or even increased risk of mortality [7–10]. Brain function alteration may occur long before any neurological symptoms are observed. Thus, to clarify the brain dysfunctions in ESRD patients is crucially important to promote our understanding of the neuropathologic mechanism of their neurological and psychiatric complications.

In the past, noninvasive neuroimaging techniques played an important role in uncovering the structural and functional abnormalities of the neural system in ESRD patients. Conventional computed tomography (CT), cerebral magnetic resonance images (MRI) and some advanced MRI techniques, such as voxel-based morphometry (VBM), diffusion tensor imaging (DTI), and perfusion-weighted imaging (FWI) were usually used to assess structural abnormalities of the brain, which included neurological complications or cerebral changes that did not reveal any abnormalities using a conventional brain MRI [11–18]. On the other hand, resting-state functional MRI (re-fMRI) has been developed as a new branch to explore the deficits of neurological dysfunction through detecting spontaneous brain activity fluctuations of patients. With this technique, some studies found functional alteration in particular areas in ESRD patients, such as decreased ReHo in the areas related to cognitive function, such as the bilateral frontal, parietal, and temporal lobes [19–21]; other work identified spatially specific disruption of functional connectivity in the default-mode network areas such as the PCC in the left middle temporal gyrus, the right anterior cingulate gyrus, and the bilateral medial superior frontal gyrus [22]. Furthermore, some other studies found the functional connectivity was impaired in the amygdala-prefrontal-PCC-limbic circuits in depressed ESRD patients and found a negative correlation between the FC of the amygdala-ACC and a reaction delay during the Stroop test, which disappeared after controlling for depression scores; the results indicated abnormal interaction between depressive mood and cognitive control deficits in ESRD patients [23,24]. In recent years, some research proposed that the salience network (SN), which is represented by the anterior insula, plays both a direct and indirect role in attention, cognition and behavioral control [25], but little is known about whether the neurological dysfunction and psychological disorders of ESRD patients are associated with the abnormality of their SN.

More recently, global functional connectivity density (gFCD) has been established to examine the density distribution of whole-brain resting-state FC without any prior hypothesis [26,27], which has been regarded as a reliable and effective technique in the analysis of resting state data in multiple neurological and psychiatric disorders including Parkinson’s disease, schizophrenia and depressive disorder [28–31]. However, it is unknown whether gFCD is altered in the brains of ESRD patients.

In the current study, we characterized and compared the gFCD differences between ESRD patients and healthy control subjects using R-fMRI, and then we examined the relationship between their alteration of gFCD and clinical markers and dialysis-related indicators. Based on previous studies, ESRD patients suffered from high prevalence of cognition dysfunction and psychological disorders, while there are strong links between the insula and ACC of the salience network and the high-level cognitive control, perception of bodily state and the experience of emotion. We hypothesized that ESRD patients could have early-stage alteration of brain functional connectivity density in their cognitive control and emotion perception-related regions, especially in the insula and ACC, compared with healthy controls. Additionally, the disrupted functional connectivity density was related to their physiological status and therapeutic conditions.

## Materials and methods

### Subjects

This study was approved by the Research Ethics Committee of both the Ninth Hospital of Chongqing and the Brain Imaging Center of Southwest University, in accordance with the Declaration of Helsinki. Written informed consent was obtained from all participants prior to data collection. Thirty ESRD patients undergoing hemodialysis were recruited, and their dialysis logs, patient rosters and hospital records were checked as information sources. There are 14 women, 16 men, with a mean age of 38.33±7.44 years, a mean dialysis duration of 51.8±30.4 months, 28 of the patients used arteriovenous fistula, and 2 patients used arteriovenous graft as their vascular access. All of the patients used polysulfone dialyzer with surface area range from 1.3 to 1.6 m^2^ in theirs dialysis. During the month before the experiment, 76.67% of patients experienced intradialytic hypotension at least once.

All of the patients self-identified as right-handed with normal sight. The exclusion criteria were as follows: (a) psychiatric disorders or major neurologic disorders (e.g., severe head injury, stroke, epilepsy or visible lesions); (2) ischemic diseases including acute ischemic cerebrovascular disease, acute peripheral arterial occlusion, advanced liver or heart failure; (c) Montreal Cognitive Assessment,(MoCA) score<26; (d) substance abuse including drugs, alcohol and cigarettes; (e) traumatic history. Dialysis-related indicators including blood pressure fluctuations in dialysis, the delivered dialysis dose measured single-pool kt/V(spKt/V), and ultrafiltration volume of dialysis of each ESRD patient were obtained from the patient’s electronic medical records, which were calculated by averaging values for 3 consecutive months prior to MR imaging. Laboratory values from ESRD patients including hemoglobin level, serum albumin level, blood urea level, serum phosphate, serum calcium, and parathyroid hormone level were measured in ESRD patients within 24 h before MR examination.

Thirty healthy right-handed controls with normal sight (13 men, 17 women, mean age 39.17±5.7 years) were recruited from the local community. All of the healthy controls had no kidney or liver disease or other systemic disorders such as hypertension and diabetes. Other exclusion criteria were similar to those of the ESRD patients.

All subjects were assessed by the Montreal Cognitive Assessment (MoCA) and the Beck Depression Inventory (BDI) before MR examination.

### MRI data acquisition

Functional imaging data were acquired using gradient-echo planar imaging (EPI) sequences with the following parameters: slices = 32, repetition time (TR)/echo time (TE) = 2000/30 ms, flip angle = 90, field of view (FOV) = 220 × 220 mm, and thickness/slice gap = 3/1 mm, and voxel size = 3.4 × 3.4 × 4 mm^3^. During the resting-state MRI scanning, the subjects were instructed to lie down, close their eyes, rest without thinking about a specific thing, and refrain from falling asleep. The total duration of resting-state MRI scanning was 8 minutes and 4 seconds, including 242 contiguous whole-brain resting-state functional images. Additionally, individual high-resolution anatomical images were also acquired using a T1-weighted three-dimensional volumetric magnetization-prepared rapidly acquired gradientecho sequence with the following parameters: slices = 176, TR/ TE = 1900/2.52 ms, flip angle = 7, field of view (FOV) = 256× 256 mm, and thickness/slice gap = 1/0 mm, and voxel size = 1.0 × 1.0 × 1.0 mm3.

### MRI data processing

The resting-state data were processed using the toolbox for Data Processing & Analysis of Brain Imaging (DPABI) [32], which is based on the SPM8 software package (https://www.fil.ion.ucl.ac.uk/spm/software/spm8/). To account for signal equilibrium and the participants’ adaptation to their immediate environment, the first 10 volumes of the functional images were deleted. Then, slice timing and head motion correction were conducted for the remaining time points. Covariates were regressed out from the time series of each voxel, which included head motion, white matter signal and cerebrospinal fluid signal [33]. In addition, the Friston 24-parameter model (6 motion parameters, 6 temporal derivatives and the 12 corresponding squared items) was used to regress out head motion effects [34]. Filtering with a bandpass filter (0.01–0.1 Hz) was chosen to reduce the effects of low-frequency drift and high-frequency noise. The registered images were spatially normalized to the Montreal Neurological Institute (MNI) template The images were resampled to 3-mm cubic voxels and smoothed using a 6-mm full-width at half maximum Gaussian kernel. Additionally, the scrubbing procedure was conducted, excluding any volume with a framewise-dependent value exceeding 0.5, with the two subsequent volumes and one preceding volume [2]. Finally, normalization quality was monitored by checking the normalization image subject by subject.

### Functional connectivity density

We used the DPARSF toolbox to calculate the functional connectivity density (FCD) of each voxel. Increased FCDs represent increased number and strength of the respective FC, indicating its significance in the brain. Pearson’s correlation coefficients were computed between all pairs of brain voxels so that the whole-brain functional connectivity matrix for each participant could be constructed. The degree centrality maps were computed using 0.4 as the threshold for determining edges [26]. Thus, whole-brain maps were derived by counting the number of voxels where the correlation between a voxel with another voxel in the BOLD time series exceeded the threshold in whole-brain binarized graph maps.

### Statistical analysis

A two-sample t-test was chosen to examine the group differences in gFCD between ESRD patients and healthy control groups in a voxel-wise manner using a general linear model with age, gender, and education as nuisance covariates. A correction for multiple comparisons was performed using p < 0.05 with familywise error (FWE) corrected at the voxel level.

A partial correlation analysis was used to assess the relationships between FCD and neuropsychological measures, and clinical variables (MoCA scores, BDI scores, hemoglobin, albumin, urea, calcium, phosphate, parathyroid hormone, spKt/V, systolic pressure fluctuations in dialysis, diastolic pressure fluctuations in dialysis, hemodialysis duration and ultrafiltration volume) in the ESRD group after controlling for age, gender and education.

## Results

### Demographic and clinical measures

A total of 60 participants entered the study. The demographic and clinical data of ESRD patients were presented in Table 1. There were no significant differences in age (*P* = 0.63), gender (*P* = 0.80), MoCA (*P* = 0.29) or education (*P* = 0.25) between the two groups. Significantly higher BDI scores were observed in ESRD patients compared with healthy controls (HCs) (*P*<0.001). The mean hemoglobin, albumin, urea, calcium, phosphate, parathyroid hormone, spKt/V, systolic pressure fluctuations in dialysis, diastolic pressure fluctuations in dialysis and ultrafiltration volume for patients were 121.67±17.94 g/L, 41.26±2.80 g/L, 24.23±5.37 mmol/L, 2.54±.20 mmol/L, 1.74±.54 mmol/L, 53.73±63.97 pmol/L, 1.35±.24, 18.15±3.53 mmHg, and 14.06±3.35 mmHg, 2.22±0.54 L, respectively.

**Table 1:** The demographic and clinical data of ESRD patients and controls.

Variable	ESRD patients	Healthy controls	*P* value
Age (years)	38.3±7.4	39.2±5.7	0.63^a^
Sex (Male/Female)	14/16	13/17	0.80^b^
MoCA (score)	27.9±1.0	28.0±1.1	0.29^a^
Visuospatial / Executive	4.7±0.5	4.7±0.5	1.00 ^a^
Naming	2.9±0.3	2.9±0.3	1.00 ^a^
Attention	1.9±0.3	1.9±0.3	1.00 ^a^
Digit span	.9±.2	0.9±0.2	1.00 ^a^
Calculation	2.9±0.3	2.8±0.5	0.51 ^a^
Verbal (Fluency& Repeting)	2.8±0.4	2.8±0.4	1.00 ^a^
Abstraction	1.5±0.5	1.7±0.4	0.06 ^a^
Recall	4.0±0.7	4.0±0.6	0.84 ^a^
Orientation	6.0±0.0	6.0±0.0	
Education(years)			
1–6 years	3	2	0.25 ^b^
7–12 years	25	23	
>15 years	2	5	
BDI (score)	9.1±9.2	3.2±3.2	0.00^a^
S. hemoglobin (g/L)	121.7±18.0	N/A	-
S. albumin (g/L)	41.3±2.8	N/A	-
S. urea (mmol/L)	24.2±5.4	N/A	-
S. calcium (mmol/L)	2.5±.2	N/A	-
S. phosphate (mmol/L)	1.7±.5	N/A	-
Parathyroid hormone (pmol/L)	53.7±64.0	N/A	-
Dialysis duration (month)	51.8±30.4	N/A	-
spKt/V	1.4±.2	N/A	-
Systolic pressure fluctuations in dialysis (mmHg)	18.2±3.5	N/A	-
Diastolic pressure fluctuations in dialysis (mmHg)	14.1±3.4	N/A	-
Ultrafiltration volume (L)	2.22±0.54	N/A	-

All quantitative data are expressed as the mean±standard deviation; numbers for sex data

MoCA Montreal Cognitive Assessment; BDI Beck Depression Inventory.

^a^ The *P* value was obtained by two-sample *t* test

^b^ The *P* value was obtained by chi-square test

N/A = not applicable

### FCD results

**Fig 1:**
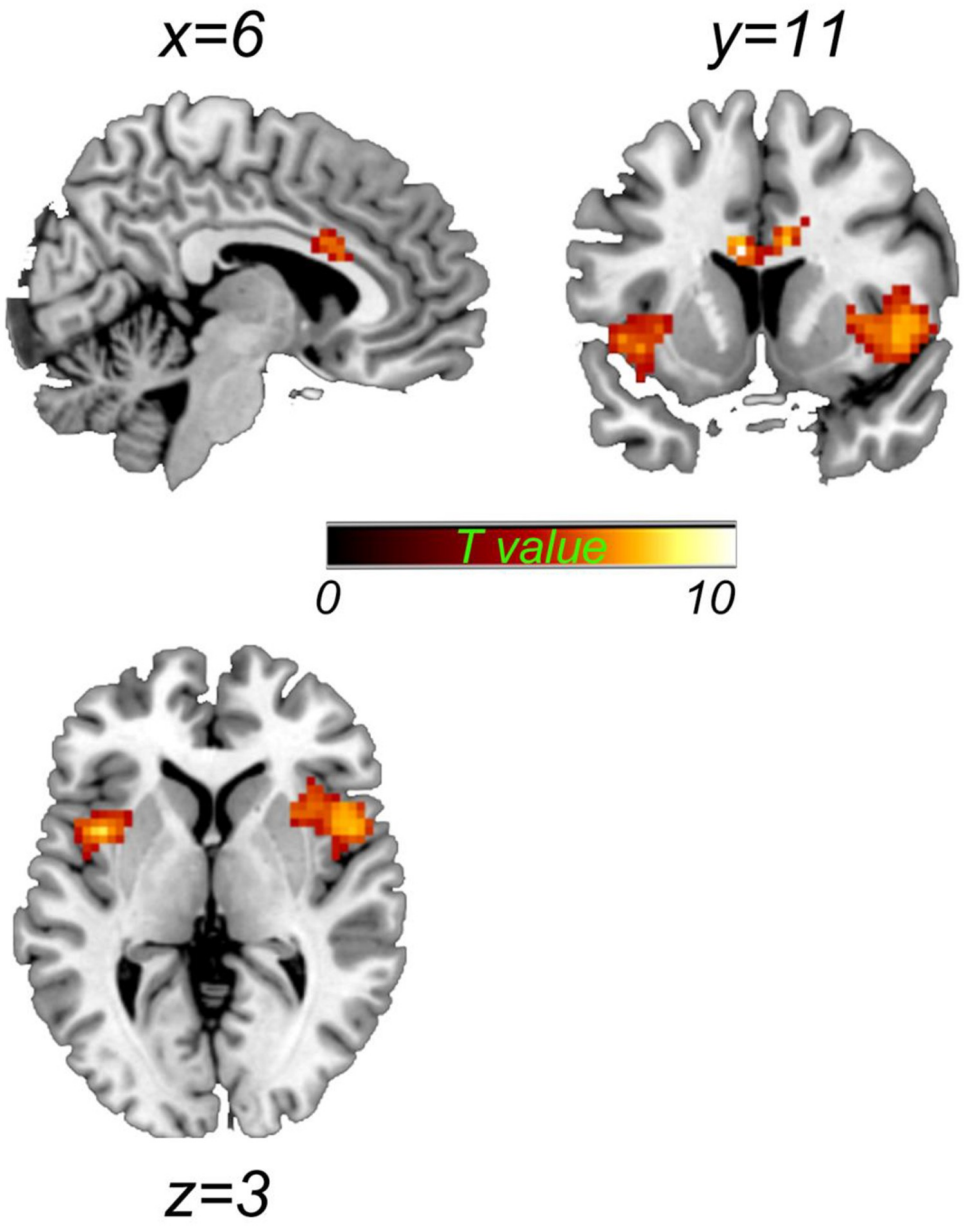
Group difference of global functional connectivity density (gFCD) map between experimental and control groups (P<0.05, corrected for family-wise error (FWE), voxel number > 50). Patients showed significantly increased gFCD in the bilateral insula and dorsal anterior cingulated cortex (dACC) (warm color) compared to controls.

Using the two-sample test method with age, gender, and education as the covariates, compared with the healthy controls, the ESRD patients exhibited a significantly increased gFCD in the bilateral insula and dorsal anterior cingulated cortex (dACC) (all *P* < 0.05 with familywise error corrected, Fig 1, Table 2). No regions showed decreased gFCD under the same statistical threshold. Furthermore, we extracted grey matter volume of the brain regions showing significant different gFCD between ESRD patients and healthy control group, and did correlation analysis between gFCD values and structural mean grey matter volume for every cluster. There were no significant correlations between gFCD and structural mean grey matter volume for every cluster (dACC: r = 0.32, p = 0.10; left insula: r = 0.02, p = 0.91; right insula: r = -0.135, p = 0.50).

**Table 2:** Significant difference between the two groups in global functional connectivity density (FCD).

	Regions	MNI coordinates (x,y,z)			Voxels	Peak value (t)
**ESRD VS HC** (Increased)						
	Left Insula	-39	6	3	151	9.13
	Right Insula	45	15	-6	253	8.28
	dACC	9	12	27	106	8.49

Results are *P*<0.05, corrected for family-wise error (FWE), voxel number > 50. Dorsal anterior cingulated cortex (dACC); end-stage renal disease (ESRD); healthy controls (HC).

### Relationship between FCD and clinical variables

**Fig 2:**
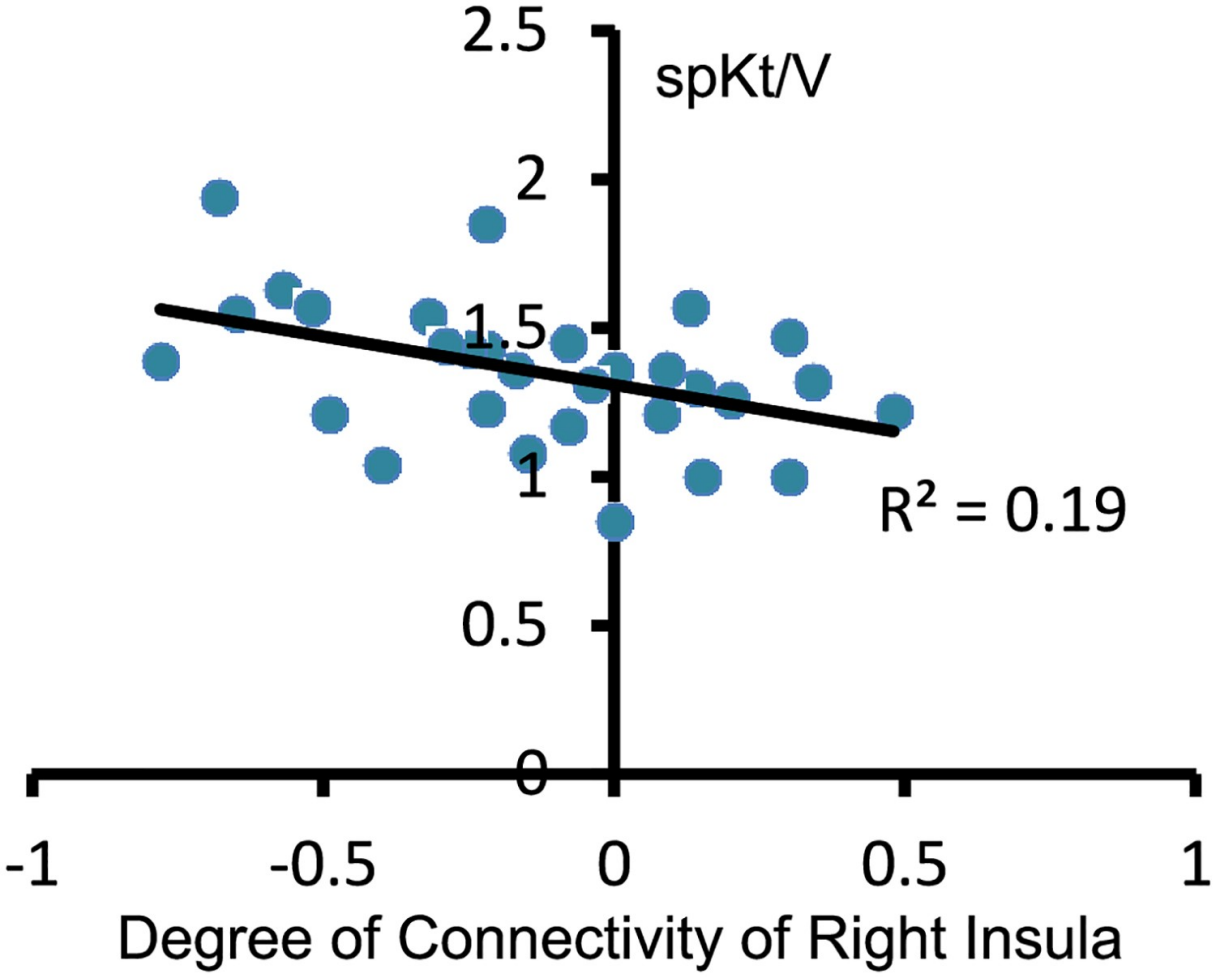
Correlation analysis between FCD of the right insula and spKt/V in ESRD patients (r = 0.44, *P*< 0.05). spKt/V, single-pool kt/V.

Significant correlations were observed between the degree of connectivity of the right insula and spKt/V only (r = 0.44, *P*< 0.05, Fig 2). No significant correlations were found between any regional FCD and other clinical variables (all *P*>0.05).

## Discussion

The aim of the present study is to investigate and characterize the functional connectivity density of ESRD patients on routine hemodialysis compared to healthy controls. We found that ESRD patients had hyperactivity in both the bilateral insula and dACC compared with pairwise matched healthy controls. Additionally, we found that the spKt/V of ESRD patients is negatively correlated with increased activation of the right insula.

Seeley et al. demonstrated the existence of an independent brain network comprised of the AI and dorsal ACC, along with subcortical structures including the amygdala, the substantia nigra/ventral tegmental area, and the thalamus; they also were the first to refer to this component as the “salience network” in 2007, proposing its function of integrating highly processed sensory data with visceral, autonomic and hedonic “markers” to guide behavior [35]. Converging evidence from a number of brain imaging studies suggests that the AI and ACC form the core of the SN and help to generate a state of heightened physiological awareness of salient stimuli, whether cognitive, homeostatic, or emotional, which have a major impact on how a stimulus is subsequently processed [25,36,37]. There is also increasing evidence showing that abnormal activation in the hub of the SN was often associated with subsequent pathology results. It has been reported that greater task-related insula activation was associated with higher levels of anxiety proneness and a greater tendency of neuroticism [38,39]. In addition, studies indicate that greater insula activation was associated with depression and insomnia, as hyperactivity of the insula may underlie elevated susceptibility to experiencing negative emotion or subthreshold negative emotion, such as depression, anxiety, worry and rumination [40,41]. Here, we also found a hyperactive functional connectivity density in the SN in ESRD patients compared with healthy controls, and those study results above provided us new idea for understanding why ESRD patients suffered from higher prevalence of depression, anxiety and symptom burden. In our study, 15 out of 30 ESRD patients suffered from depressed mood, and the BDI scores of the patients is significantly higher than in the healthy controls. Thus, the abnormal activity of the salience network may relate to frequent negative emotional arousal. Meanwhile, in the present study, we did not find a significant correlation between the BDI scores and insula activation, possibly due to the insula being an important hub in a salience network, and the depressive symptoms may correlate with functional connectivity between the insula and specific brain areas. Further studies are required to clarify the relationship between the functional connectivity of the insula and emotional arousal in ESRD patients.

We further examined the correlation between the FCD and the demographic, clinical data of the patients with ESRD and found negative correlation between the FCD of the right insula and spKt/V, suggested that hemodialysis adequacy may play an important role in decreasing the hyperactivity of insula in ESRD patients. ESRD patients usually have a high level of uremic toxin in particular urea, which is believed to be an important risk factor for brain dysfunction [42–44]. The National Cooperative Dialysis Study demonstrated that increased fractional removal of urea during the dialysis session reduced electroencephalographic abnormalities in patients receiving maintenance hemodialysis [45]. However, no significant correlations were found between any regional FCD and other clinical variables including urea. This maybe because of the individual difference of the tolerance to urea in patients, and spKt/V is determined also by other variables including time of dialysis in hours, the ratio of the ultrafiltrate volume removed to the post-dialysis weight. Previous research suggested that cerebral perfusion in hemodialysis patients oscillates during the interdialytic cycle, and may contribute significantly to the etiology of the observed cerebral atrophy, cognitive deficits couple with other factors [46]. So the relative blood volume changes during HD may impact on cerebral perfusion and indirectly on their brain functional connectivity.

There are limitations of the current study that should be addressed in future investigations. First, unfortunately, we did not manipulate any trait anxiety or neuroticism assessment, and we did not give patients any Structured Clinical Interviews for DSM-IV to clarify the presence of their “fragile status”. In future studies, examination should include not only fMRI but should also ascertain the presence of subthreshold feeling or mood with assessment, structured clinical interview or physiological measures such as skin conductance. Second, inevitably, there are other complications such as hypertension, anemia, and hyperparathyroidism in the ESRD patients in our study. We cannot exclude the possibility that the abnormal activity in the SN is the consequence of the comorbidities. More homogeneous and purer samples should be recruited to clarify this issue in future research. Third, unlike the previous study [22] on ESRD patients, we did not find possible functional alterations related to cognitive impairment, which might be because of the difference in neuropsychological tests we used to screen the patients. We chose MoCA instead of MMSE, which is believed superior for detection of mild cognitive impairment [47–51], to screen patients with normal global neurocognition. Fourth, all patients in this study experience a long-term hematodialysis. Previous studies suggest that a long-term hematodialysis can result in significantly cerebral abnormalities of oxygenation [46] and cerebral blood flow [52–54] in ESRD patients, which can affect the cerebral circulation and brain function[55–57]. Therefore, the findings should be interpreted with caution and further study is needed to collect data from patients with chronic kidney disease (stage 4–5) without hemodialysis to be able to explore whether and how hemodialysis itself can affect the intrinsic functional connectivity. Fifth, previous revious studies indicated that abnormal long- and short-range functional connectivity density in physical disease [58,59], in the future study, we are planning to investigate the alternation of brain structural and functional hub in ESRD patients by recruiting a large sample. Lastly, due to the cross-sectional design of our study, we cannot address how the vulnerability of the patients progresses to symptom burden and if there is any possible link between the vulnerability and their risk of cognitive impairment. Future longitudinal studies would provide insights into this issue.

## Conclusions

In summary, we found the neurologically asymptomatic ESRD patients exhibited hyperactivity in the bilateral insula and dACC, which are core components of the “salience network”. The enhanced activation of the right insula of the patients was negatively correlated with their spKt/V, suggesting that hemodialysis adequacy may play an important role in decreasing the pathologic activity of insula in ESRD patients.
